# The flavagline FL3 interferes with the association of Annexin A2 with the eIF4F initiation complex and transiently stimulates the translation of *annexin* A2 mRNA

**DOI:** 10.3389/fcell.2023.1094941

**Published:** 2023-05-12

**Authors:** Ann Kari Grindheim, Sudarshan S. Patil, Canan G. Nebigil, Laurent Désaubry, Anni Vedeler

**Affiliations:** ^1^ Department of Biomedicine, Faculty of Medicine, University of Bergen, Bergen, Norway; ^2^ Regenerative Nanomedicine Laboratory (UMR1260), Faculty of Medicine, FMTS, INSERM-University of Strasbourg, Strasbourg, France

**Keywords:** Annexin A2, FL3, translation, initiation complex, eIF4F

## Abstract

**Introduction:** Annexin A2 (AnxA2) plays a critical role in cell transformation, immune response, and resistance to cancer therapy. Besides functioning as a calcium- and lipidbinding protein, AnxA2 also acts as an mRNA-binding protein, for instance, by interacting with regulatory regions of specific cytoskeleton-associated mRNAs.

**Methods and Results:** Nanomolar concentrations of FL3, an inhibitor of the translation factor eIF4A, transiently increases the expression of AnxA2 in PC12 cells and stimulates shortterm transcription/translation of anxA2 mRNA in the rabbit reticulocyte lysate. AnxA2 regulates the translation of its cognate mRNA by a feed-back mechanism, which can partly be relieved by FL3. Results obtained using the holdup chromatographic retention assay results suggest that AnxA2 interacts transiently with eIF4E (possibly eIF4G) and PABP in an RNA-independent manner while cap pulldown experiments indicate a more stable RNA-dependent interaction. Short-term (2 h) treatment of PC12 cells with FL3 increases the amount of eIF4A in cap pulldown complexes of total lysates, but not of the cytoskeletal fraction. AnxA2 is only present in cap analogue-purified initiation complexes from the cytoskeletal fraction and not total lysates confirming that AnxA2 binds to a specific subpopulation of mRNAs.

**Discussion:** Thus, AnxA2 interacts with PABP1 and subunits of the initiation complex eIF4F, explaining its inhibitory effect on translation by preventing the formation of the full eIF4F complex. This interaction appears to be modulated by FL3. These novel findings shed light on the regulation of translation by AnxA2 and contribute to a better understanding of the mechanism of action of eIF4A inhibitors.

## Graphical Abstract

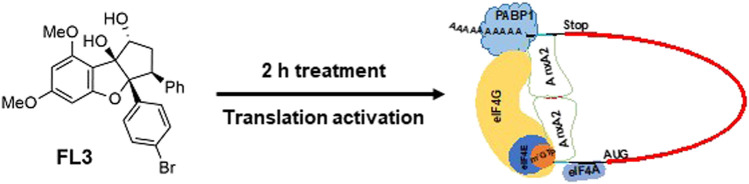

## 1 Introduction

Annexin A2 (AnxA2) belongs to a family of structurally related, calcium-dependent anionic phospholipid-binding proteins, which are present in virtually all eukaryotic cells (Dreier et al., 1998; Gerke and Moss, 2002). AnxA2 is a multi-functional and -compartmental protein possessing a variety of cellular functions, related to cell proliferation, membrane-cytoskeleton interactions, endo- and exocytosis, as well as mRNA transport and translation (Gerke and Moss, 2002; Gerke et al., 2005; Vedeler et al., 2012; Grindheim and Vedeler, 2016; Gabel et al., 2020). Moreover, it functions in the biogenesis of exosomes, small vesicles derived from multivesicular bodies in the endocytic pathway (Valapala and Vishwanatha, 2011; Grindheim and Vedeler, 2016; Hessvik and Llorente, 2018), which are secreted by many cell types, including neuronal cells. AnxA2 undergoes numerous post-translation modifications (PTMs), which change its affinity for different ligands and in turn discriminate between its different functions (Lauvrak et al., 2005; Vedeler et al., 2012; Grindheim et al., 2017; Gabel et al., 2019).

Upregulation of AnxA2 is generally associated with an aggressive and metastatic cancer phenotype, as well as resistance to chemotherapy, being directly related with advanced clinical stages of several cancer types such as lung, breast and colorectal tumors (Xu et al., 2015) as well as neuronal malignancies (Maule et al., 2016; Christensen et al., 2018). On the other hand, an inverse correlation was identified in the case of esophageal carcinomas and head and neck squamous cell carcinomas, where the clinical stage advancement, more frequent recurrence and both regional lymph node and distant metastasis are all closely related with the downregulation of AnxA2 (Xu et al., 2015). Regarding the immune system, the upregulation of AnxA2 has been reported to stimulate the production of TNF-α, IL-1β and IL-6 as well as other chemokines to promote inflammation (Swisher et al., 2007). AnxA2 peptides presented by MHC class II-positive cancer cells can also activate antigen-specific T cells and thus produce an immune response that is potentially useful in immunotherapy (Heinzel et al., 2001; Zheng and Jaffee, 2012; Weyd, 2016). Additionally, in response to oxidative stress, IL-1α and AnxA2 colocalize at the plasma membrane (PM) in epithelial cells to communicate with neighboring cells (Novák et al., 2020). Knock-out of AnxA2 in mouse enhances activation of the NLR family pyrin domain containing 3 (NLRP3) inflammasome in dendritic cells (Scharf et al., 2012). Thus, it has been suggested that AnxA2 acts as a key endogenous factor in reducing the pro-inflammatory response after acute brain injury (Liu et al., 2019). These findings indicate that sustained high levels of AnxA2 are largely associated with adverse effects, while transient short-term upregulation of the protein may be beneficial regarding immune stimulation and protection against oxidative stress.

**FIGURE 1:**
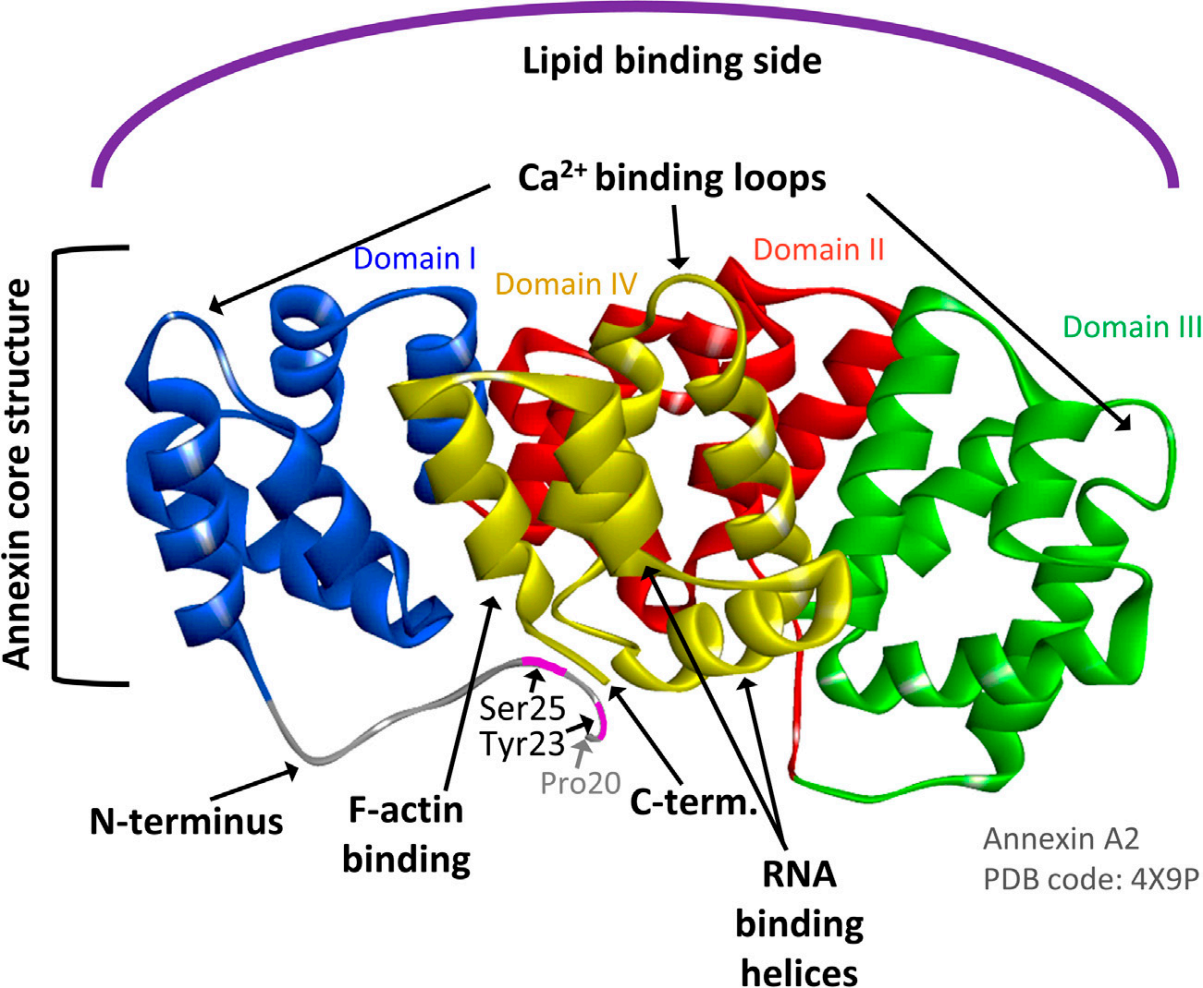
AnxA2 structure indicating the Ca^2+^, RNA, actin and membrane binding sites of the protein. Ribbon representation of the crystal structure of AnxA2 (pdb: 4X9P) in which Pro20 is the first visible amino acid (counting the first Ser as amino acid 1). Domains I, II, III, and IV which are part of the structural core of AnxA2 are shown in blue, red, green and yellow, respectively. The Tyr23 and Ser25 phosphorylation sites in the N-terminus are also indicated. Modified from (Grindheim et al., 2017).

**FIGURE 2:**
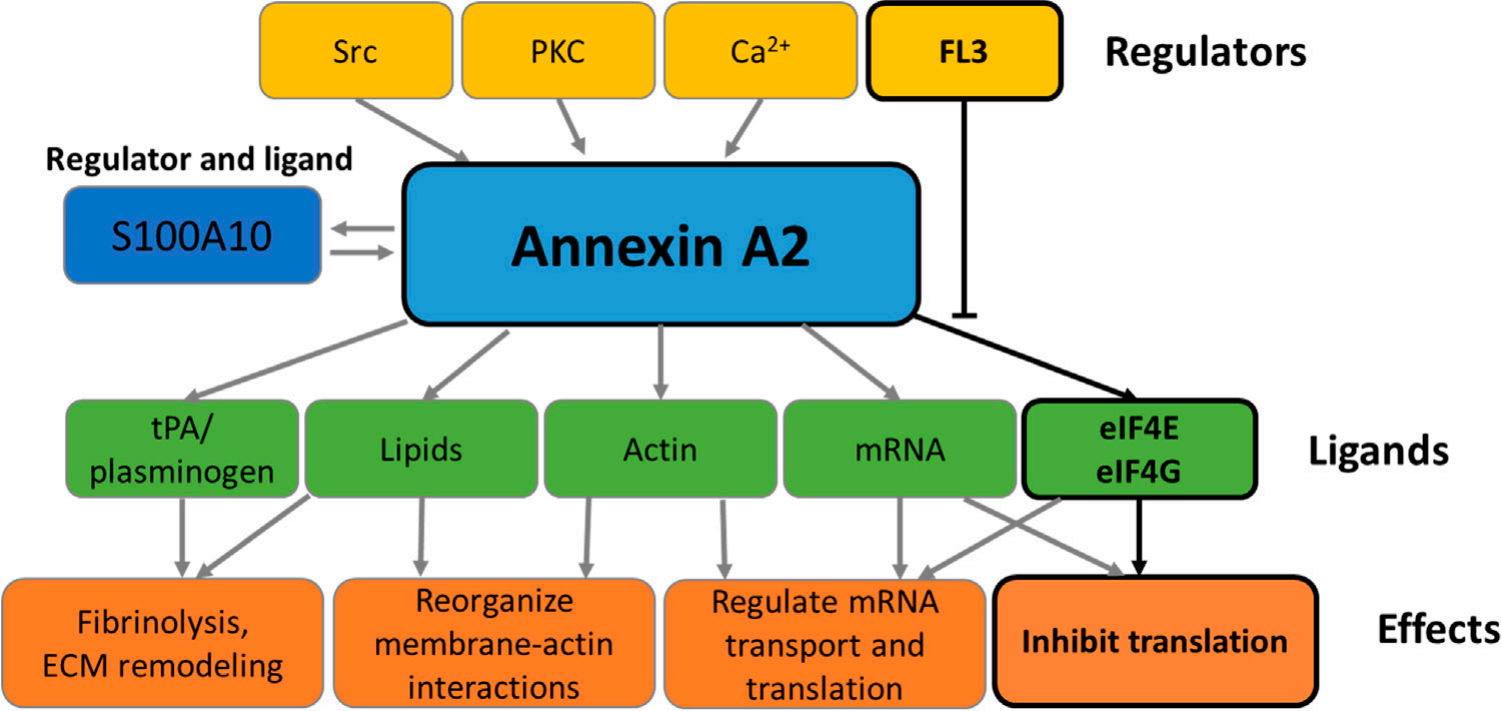
Functions of Annexin A2. The schematic representation summarizes the effectors and functions of AnxA2 as detailed in the Introduction. The boxes framed in black refer to the effect of FL3 on the regulation of AnxA2 on translation of its cognate mRNA.

The expression of AnxA2 is under the control of numerous signaling pathways and varies between different cells and tissues (Xu et al., 2015). Thus, this multifunctional protein may display distinct major functions depending on the cell type. The regulation of AnxA2 expression is very complex since its functional repertoire is strictly regulated by ligand binding, subcellular localization, and a variety of PTMs (Grindheim et al., 2017). AnxA2 has numerous interacting ligands. One of the main ligands of AnxA2 is S100A10 which is important for its association with membranes (Gerke and Moss, 2002) and thereby also functions as an effector (Figures 1, 2). Another important ligand is actin, as AnxA2 is known to participate in the regulation of actin dynamics (Figures 1, 2) (Hayes et al., 2006). Both ligands are important in the context of the suggested roles of AnxA2 in tumor progression.

Thus, AnxA2 participates in various cellular processes through its interactions with other signaling proteins and lipids (Figure 2) (Huang et al., 2022). Extracellularly, AnxA2 in complex with S100A10 functions as a regulator of hemostasis by facilitating the activation of plasminogen to plasmin (Lim and Hajjar, 2021). AnxA2 has been implicated in the regulation of the phosphatidylinositol 3-kinase (PI3K)/Akt signaling pathway, which is linked to cell survival, proliferation, and migration (Chen et al., 2018). AnxA2 binds Ca^2+^ and regulates calcium signals, which are involved in various cellular processes, such as apoptosis, cell differentiation, and neurotransmitter release (Gerke et al., 2005). AnxA2 interacts with the Rho-associated protein kinase (ROCK) pathway, which is connected to cytoskeleton remodeling and cell migration (de Graauw et al., 2008; Rescher et al., 2008). Furthermore, AnxA2 has been implicated in the regulation of the inflammatory response through its interaction with pro-inflammatory signaling molecules such as nuclear factor-kappa B (NF-κB) (Chen et al., 2022).

AnxA2 is phosphorylated by Src kinase at Tyr23 (counting the first Ser as amino acid no 1) (Figure 1) (Grindheim et al., 2017). It interacts with receptor of activated protein kinase C 1 (RACK1), which appears to mediate the interaction of Src with AnxA2 and thereby facilitate its Tyr23 phosphorylation (Fan et al., 2019). Interestingly, the binding of RACK1 to ribosomes is important for the recruitment of the initiation factor eIF4E and therefore crucial for efficient translation of capped mRNAs (Gallo et al., 2018). Tyr23 phosphorylation of AnxA2 is also involved in the regulation of actin dynamics via various signaling pathways and inhibits the ability of the AnxA2-S100A10 complex to bind and bundle actin filaments (Figure 2). The phosphorylation of AnxA2 at Ser11 dissociates the AnxA2-S100A10 heterotetrameric complex while Ser25 phosphorylation is related to membrane binding (Grindheim et al., 2017) and sequestration of translationally inactive mRNP complexes in combination with other post-translationally modifications (Figure 2) (Aukrust et al., 2017). It has been suggested that Ser25 phosphorylation stabilizes a specific conformation of AnxA2 in which the binding sites for mRNA and G-actin in domain IV of the core structure become more accessible (Figure 1) (Hayes et al., 2009; Grindheim et al., 2017).

In addition to their many favorable pharmacological activities, flavaglines have shown promising anticancer properties (Nebigil et al., 2020; Greger, 2022). The flavagline FL3 is a synthetically modified Br derivate of rocaglaol and demonstrates higher cytotoxicity against several cancer cell lines than its mother compound (Thuaud et al., 2009). FL3 targets the initiation factor eIF4A, a helicase in the eIF4F complex by promoting the formation of a stable eIF4A-RNA complex (Boussemart et al., 2014). It thus clamps the eIF4A onto the mRNA and hampers the formation of new eIF4F complexes by preventing free eIF4E to re-enter new eIF4F complexes (Bordeleau et al., 2008). Preferentially, mRNAs with highly structured 5′UTRs and/or containing polypurine-rich sequences are affected by the flavaglines (Wolfe et al., 2014; Shen and Pelletier, 2020). These mRNAs form a stable RNA-flavagline-eIF4A complex that blocks the scanning of the 40 S ribosomal subunit. The eIF4A initiation factor has also received attention as a putative target for anticancer drugs, since it is upregulated in many cancers (Malka-Mahieu et al., 2017). In addition, the overexpression of eIF4E and eIF4G of the eIF4F complex has also been correlated with malignant progression and poor prognosis in various cancers (Mamane et al., 2004; Pelletier et al., 2015). Here we present evidence that AnxA2 transiently associates with the eIF4F complex by interacting with the eIF4E, eIF4G and also the poly(A)-binding protein (PABP1) explaining the inhibition of AnxA2 on translation. A more stable interaction is RNA-dependent. *In vitro* translations, FL3 is able to partly relieve the inhibitory effect of AnxA2 on translation.

## 2 Materials and methods

### 2.1 Culture and treatment of PC12 cells

The rat adrenal pheochromocytoma (PC12) cells, representing a readily adherent sub-clone derived from the original PC12 cell line (Greene and Tischler, 1976) (a generous gift from Prof. Jaakko Saraste, University of Bergen, Norway), were grown in Gibco RPMI 1640 medium (ThermoFisher Scientific, Waltham, United States) supplemented with 10% (v/v) heat-inactivated horse-serum, 5% (v/v) foetal bovine serum, 2 mM L-glutamine, 100 units penicillin/mL and 100 µg streptomycin/mL. All supplements were from Sigma-Aldrich (Saint-Louis, United States). Cells were recently authenticated and routinely tested for contamination. As described previously (Grindheim et al., 2014), the cells were routinely cultured at 37°C in a humidified atmosphere of 21% O_2_ supplemented with 5% CO_2_. A 10 mM stock of FL3 in DMSO was further diluted to 20 nM (or the other indicated concentrations) in the complete RPMI 1640 culture medium before incubation with the cells. Actinomycin D (Act D; 4 μg/mL; A9415; Sigma-Aldrich, Saint-Louis, United States) and cycloheximide (CHX) (10 μg/mL; 239764; Sigma-Aldrich, Saint-Louis, United States) were added to the medium for 30 min (Mackinnon et al., 2012) before the addition of FL3 and incubation was continued for further 2 h (Figure 4D) or CHX and Act D were added simultaneously with FL3 (Figures 4A–C).

### 2.2 Cell fractionation and lysates

A total PC12 cell lysate was obtained by incubation for 15 min in Lysis buffer (50 mM Hepes, 150 mM NaCl, 1 mM EDTA, 0.5% (w/v) NP-40, 1 mM dithiothreitol, 1 mM Na_3_VO_4_, 1 mM NaF; all from Sigma-Aldrich, Saint-Louis, United States) supplemented with 1× protease inhibitor cocktail (EDTA-free; 11836170001; Roche, Basel, Switzerland) and centrifuged for 20 min at 12 000 g at 4°C. The cytoskeletal fraction of PC12 cells was obtained essentially as described previously (Vedeler et al., 1991; Aukrust et al., 2017). In essence, after preparation of the cytosolic fraction, the cytoskeletal fraction was isolated following a 20 min incubation at room temperature in 130 mM KCl buffer (130 mM KCl, 5 mM MgSO_4_, 70 µM CaCl_2_, 8.6% sucrose, 10 mM Triethanolamine; pH 7.4) supplemented with the above-described 1 × protease inhibitor cocktail. The isolation of cytoplasmic and nuclear fractions was carried out according to the protocol provided in the “NE-PER^®^ Nuclear and Cytoplasmic Extraction Reagents” kit (ThermoFisher Scientific, Rockford, United States). Fractionation of the cytoplasm and harvesting of mitochondria was carried out using the protocol (option A) provided in the ‘Mitochondria Isolation Kit for Cultured Cells’ (ThermoFisher Scientific, Rockford, United States) as described previously (Aukrust et al., 2017).

### 2.3 Protein determination by the bicinchoninic acid (BCA) method

The BCA protein assay was used for quantitation of total protein in lysates or subcellular fractions using BSA as a protein standard. The procedure was carried out according to the manual in the kit (23225, Pierce, ThermoFisher Scientific, Waltham, United States).

### 2.4 Immunofluorescence microscopy

PC12 cells were grown on poly-L-Lys-coated glass coverslips and treated as indicated above. The cells were fixed, permeabilised and blocked against non-specific binding of antibodies as described previously (Grindheim et al., 2014; Grindheim et al., 2016) prior to staining with primary polyclonal antibodies against AnxA2 (1:250; ab41803, Abcam, Cambridge, UK). The bound primary antibodies were detected using appropriate DyLight-488- or DyLight-594-conjugated Fab2 fragments (1:50; Jackson ImmunoResearch Laboratories, West Grove, United States). The coverslips were inverted and mounted on objective glasses on a small drop of Vectashield mounting medium containing 4′,6-diamino-2′-phenylindole (DAPI) (Vector Laboratories, Burlingame, United States). Confocal imaging was performed using a Leica SP5 AOBS confocal laser scanning microscope equipped with 405 diode and argon and helium neon lasers (Leica Microsystems, Wetzlar, Germany). Optical sections were obtained using the 63×/1.4 NA HCX Plan-Apochromat oil-immersion objective (Leica, Wetzlar, Germany), ∼1 Airy unit pinhole aperture and appropriate filter combinations. Confocal images were obtained in Leica Application Suite (LAS) AF. Figures were made in Microsoft Publisher for the images and GraphPad Prism for the graphs. Quantitation was done with ImageJ and transferred to GraphPad.

### 2.5 7-Methyl-GTP cap pulldown assay

m^7^GTP pulldown assays have been described in detail elsewhere (Marti et al., 2017). In brief, proteins from total cell lysate or the cytoskeletal fraction were used for the cap pulldown assays as indicated in Figure 5. Thus, 300 μg protein was applied to 25 μL pre-washed γ-aminophenyl-m^7^GTP (C10-spacer)-Agarose (AC-155, Jena Bioscience, Jena, Germany) and incubated for 90 min at 4°C. Beads with m^7^GTP-bound proteins were washed three times with lysis buffer and bound proteins were eluted with Laemmli’s sample buffer and separated by SDS-PAGE and subjected to Western blot analysis. For RNase treatment, the cytoskeletal fraction was treated with 5 µL RNase A/T1 mix (EN0551; 2 mg/mL of RNase A and 5000 U/mL of RNase T1; ThermoFisher Scientific, Waltham, United States) for 30 min at 37°C before cap pulldown.

### 2.6 Holdup comparative chromatographic retention assays

The principle and procedure of the holdup method used to detect protein-protein interactions, including transient ones, have been described before in great detail (Charbonnier et al., 2006). Purified rat His-AnxA2 was bound to Ni^2+^-NTA agarose resin to saturation (Quiagen, Hilden, Germany) in 20 mM Tris-HCl buffer, pH 8.0 for 1 h at 4°C (16 µg protein/µL bedded resin beads = 400 µM), after pre-equilibration of the resin in the same buffer. The immobilised AnxA2 was washed 3 times with this buffer and twice with the 130 mM KCl buffer supplemented with the anti-protease cocktail (see above). Subsequently, the resin with immobilised His-AnxA2 protein and a control resin without bound protein were incubated with 125 µg of analyte (the cytoskeletal fraction)/50 µL resin beads whereafter 130 mM KCl buffer was added to a final volume of 100 µL liquid phase + 50 µL bedded resin beads (= 150 µL slush). This was incubated for 1 h on a rotating wheel in the cold room before aliquoting 2 identical samples (= 60 µL slush) for each experimental condition into small microBio-Spin Chromatography Columns (#7326204EDU; Bio-Rad laboratories, Hercules, United States). 27 μL of 1 M imidazole (final concentration 400 mM in liquid phase) was added to one sample marked “+” and 27 µL of 130 mM KCl buffer was added to the other sample marked “−“. The samples were incubated for 15 min on a rotating wheel in the cold room. Subsequently, the flowthroughs were collected, added 4x Laemmli sample buffer (Bio-Rad Laboratories, Hercules, United States) and heated for 5 min at 95°C before SDS-PAGE. For RNase treatment, the cytoskeletal fraction was treated with 5 µL RNase A/T1 mix (EN0551; ThermoFisher Scientific, Waltham, United States) for 30 min at 37°C before incubation with AnxA2 bound to the Ni^2+^ resin.

### 2.7 *In vitro* transcription and prediction of secondary structure of *anx*A2 5′UTR

The 1,356 bp form of full-length rat *anx*A2 cDNA (including sequences coding for the UTRs) (identical to NM_019905; GI: 9845233 and S73559.1) was obtained by RT-PCR using total RNA isolated from PC12 cells as previously described and cloned into the pGEM-3Zf + plasmid (Promega, Madison, United States) under the control of the T7 promoter (Aareskjold et al., 2019). By using the forward primer (5′-gaa​att​aat​acg​act​cac​tat​agg​gag​gct​ctc​tgc​aat​agg​tgc) of the rat *anx*A2 5′UTR containing the T7 promoter sequence (5′-taa​tac​gac​tca​cta​tag​gg), the rat *anx*A2 3′UTR reverse primer (5′-aaa​gta​aaa​tgg​ttt​att​c), the pGEM-3Zf + plasmid with *anx*A2 cDNA as template and the ACCUtaq DNA polymerase (Sigma-Aldrich, Merck, Darmstadt, Germany), the cDNA fragment containing a T7-promoter in front of full-length *anx*A2 was obtained. The PCR product was subjected to electrophoresis and purified from a 1% (w/v) agarose gel. The purified cDNA template was used in *in vitro* transcription assays using the HiScribe T7 High Yield RNA Synthesis Kit (New England BioLabs, Ipswich, United States) according to the manufacturer. The mRNA was extracted using the BioUltra phenol:chloroform:isoamyl alcohol (125:24:1; pH 4-5) (Sigma-Aldrich, Saint-Louis, United States) and BioUltra chloroform:isoamyl alcohol (24:1) (Sigma-Aldrich, Saint-Louis, United States) method. Subsequently, the mRNA was precipitated with ethanol (Vinmonopolet, Oslo, Norway), 0.1 volume of 3 M sodium acetate (pH 5.2; ThermoFisher Scientific, Waltham, United States) and overnight incubation at −80°C. Finally, after 70% ethanol washes, the mRNA was resuspended in double distilled water. The prediction of the secondary structure (centroid of cluster) of the rat *anx*A2 5′UTR with the lowest ΔG^o^ (37°C, 1 M NaCl, no divalent cations) was made using the sequence “gga​ggc​ucu​cug​caa​uag​gug​ccc​ggc​cca​gcu​uuu​uuu​uca​aau​g” and the Sfold program (https://sfold.wadsworth.org/cgi-bin/srna.pl) (Ding et al., 2005).

### 2.8 *In vitro* coupled transcription-translation system

The TNT^®^ T7 Quick for PCR DNA (Promega, Madison, United States) is an *in vitro* coupled transcription/translation system based on rabbit reticulocyte lysate (RRL) and was supplemented with the cDNA coding for full-length rat *anx*A2 mRNA (including the UTRs and containing a T7 promoter site; described above) and [^35^S]-Met (EasyTag™ L-[^35^S]-Met; 10 mCi/mL; PerkinElmer, Waltham, United States). AnxA2 in 20 mM Tris (pH 8) was added to the RRL before the addition of the cDNA and the RRL constituted 63% of the assays. The reaction was performed as previously described (Strand et al., 2021). Incorporated [^35^S]-Met into the AnxA2 protein and total radioactivity in the reaction mixture were measured in a Packard liquid scintillation counter.

### 2.9 *In vitro* translation system

The RRL supplemented with [^35^S]-Met (EasyTag™ L-[^35^S]-Met; 10 mCi/mL; PerkinElmer, Waltham, United States) was used for *in vitro* translations (Pelham and Jackson, 1976) of *in vitro* transcribed *anx*A2 mRNA. AnxA2 was added in the same buffer as described above and the RRL constituted 70% of the assays. Incorporation of [^35^S]-Met into AnxA2 protein was measured as described above.

### 2.10 Recombinant rat and bovine AnxA2

The coding region of rat *anx*A2 cDNA (identical to NM_019905; GI: 9845233) was obtained by RT-PCR using total RNA isolated from PC12 cells and the iScript cDNA Synthesis Kit (Bio-Rad, Hercules, United States) for the first-strand cDNA synthesis. The PCR step was performed in the presence of 2 mM Mg^2+^, as well as the AnxA2 forward (5′-atccggccatgggtatgtctactgtccacgaaatc) and reverse (5′-atccggggtacctcagtcgtcaccaccacacag) primers containing the (*Nco*I) and (*Acc*65I) (FastDigest, ThermoFisher Scientific; Waltham, United States) restriction enzyme cleavage sites (underlined), respectively. The PCR reaction was supplemented with *Pfu* DNA polymerase (Promega, Madison, United States). Subsequently, the cDNAs were cloned into the pETM10 vector (a generous gift from Dr. Gunter Stier) after restriction enzyme digestion of the PCR fragments and the plasmid with *Nco*I and *Acc*65I. Sequence verification of all clones used was performed at the Haukeland University Hospital DNA sequencing facility. His-AnxA2 was expressed overnight in BL21 bacteria at 15°C and subsequently purified on Ni^2+^-resin (Ni-NTA agarose, Qiagen, Hilden, Germany), essentially as described previously (Aukrust et al., 2006; Strand et al., 2021). The protein was gel-filtrated on a SuperdexTM 200 Increase 10/300 GL column (GE Healthcare, Chicago, United States). The purity of recombinant AnxA2 was determined by SDS-PAGE followed by Coomassie Brilliant Blue staining. The NanoDrop quantitation method was employed for purified proteins based on their extinction coefficients and the absorption was measured at 280 nm. AnxA2 was quick-frozen in liquid nitrogen and stored at −80°C. The purification of bovine His-tagged AnxA2 has been described previously (Aukrust et al., 2006; Hollas et al., 2006; Grindheim et al., 2014). For cloning of the N-terminally truncated bovine Δ20AnxA2, the same reverse primer was used as for FL AnxA2 while this forward primer was used; 5′-atccgggaagactccatgccaagtgcatacgggtcagtc containing a *Nco*I compatible cleavage site introduced by the *Bbs*I restriction enzyme type II (underlined) (FastDigest, ThermoFisher Scientific, Waltham, United States).

### 2.11 SDS-PAGE and western blot analysis

Samples from lysates and subcellular fractions were heated at 70°C for 10 min in Laemmli sample buffer (Bio-Rad Laboratories, Hercules, United States) and resolved in 10% or 4%–20% (w/v) SDS-PAGE gels. Proteins were transferred onto nitrocellulose membranes (0.2 µm pore size; (#162-0112; Bio-Rad Laboratories, Hercules, United States) by blotting performed using the Trans-Blot Turbo Transfer System (Bio-Rad Laboratories, Hercules, United States) according to the manufacturer (25 V/1.3 mA, 7 min transfer). Mouse monoclonal antibodies were used to detect AnxA2 (610069; BD Biosciences, Franklin Lakes, United States; dilution 1:1,000), tubulin (86298; Cell Signaling Technology, Danvers, United States; dilution 1:5,000), GAPDH (sc-32233; Santa Cruz Biotechnology, Dallas, United States; dilution 1:1,000) and complex II (459200; Invitrogen; Waltham, United States; dilution 1:1,000), whereas Matrix 3 (A300-591A, Bethyl laboratories, Montgomery, United States; dilution 1:1,000), AnxA2 (pSer25) (OAAF00618; Aviva Systems Biology, San Diego, United States; dilution 1:1,000) as well as eIF4A (C32B4) (#2013; dilution 1:1,000), p-eIF4E (#9741; dilution 1:1,000), eIF4E (#9742; dilution 1:1,000), eIF4G (#2498; dilution 1:1,000), PABP1 (#4992; dilution 1:1,000), and nucleolin (A300-711A, Bethyl Lab.; ThermoFisher Scientific, Waltham, United States; dilution 1:1,000) were detected by rabbit polyclonal antibodies all from Cell Signaling Technology (Danvers, United States). Primary antibody binding was followed by incubation with horseradish peroxidase (HRP)-conjugated anti-mouse antibodies or anti-rabbit antibodies (Bio-Rad Laboratories, Hercules, United States). The reactive protein bands were visualised using the WesternBright Sirius ECL HRP substrate (Advansta; San Jose, United States) and the Gel DOC XRS+ (Bio-Rad Laboratories, Hercules, United States). Densitometric analyses were performed with ImageJ software (NIH, Bethesda, United States). Densitometric values of proteins are expressed per unit of protein applied to the gel lane and normalized to loading control.

### 2.12 Statistical analysis

The arbitrary unit values are reported as mean ± SD. One-way ANOVA has been used to test repeated comparisons within the same series, and two-way ANOVA have been used for grouped analyses. We have used t-tests for comparing two groups when normality is assumed. Grubbs’ test has been used to evaluate the presence of outliers. Only values identified to be outliers with *p* < 0.05 in this test have been removed from datasets.

## 3 Results

### 3.1 FL3 transiently increases the expression of AnxA2

The rat PC12 cell line originating from a pheochromocytoma–a tumor of the adrenal medulla caused by irradiation–has been cultivated since 1976 (Greene and Tischler, 1976) and widely used as a model for neuroendocrine secretion and neuronal differentiation. Since the synthetic flavagline FL3 is a potent anticancer compound (Thuaud et al., 2009), we initially tested its effect on the viability of PC12 cells. Treatment of the cells for 24 h with 2 or 20 nM FL3 decreased their viability by about 5% and 25%, respectively, relative to untreated cells (Supplementary Figure S1).

**FIGURE 3:**
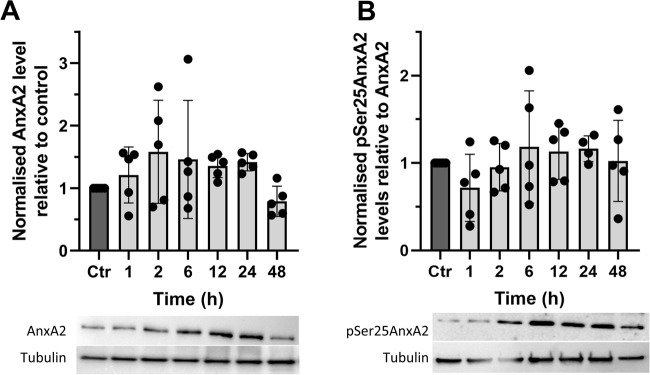
The synthetic flavagline FL3 transiently increases the expression of AnxA2 (Panel **A**) and the Ser25 phosphorylated AnxA2 form follows the level of total AnxA2 (Panel **B**). Lysates were prepared from control (Ctr) PC12 cells and after their treatment for 1, 2, 6, 12, 24, and 48 h with 20 nM FL3. 10 μg of proteins separated by 10% SDS-PAGE were transferred to nitrocellulose membranes for Western blot analysis with monoclonal antibodies against AnxA2, or polyclonal antibodies against pSer25AnxA2, with tubulin serving as a loading control, as indicated. Representative blots and the results from five independent experiments (*n* = 5) are shown. AnxA2 (Panel **A**) is expressed in arbitrary units of intensity relative to the control sample (set = 1) after normalization to the loading control tubulin while pSer25AnxA2 (Panel **B**) is expressed in arbitrary units of intensity relative to the control sample (set = 1) after normalization to total AnxA2. The standard deviations are also indicated. Statistical significance compared to control was determined by one-way ANOVA and Dunnett’s multiple comparisons test (* <0.05).

Since AnxA2 is involved in the progression of cell transformation, we next investigated the long-term effects of FL3 at nanomolar concentrations on the expression of AnxA2 in PC12 cells (Figure 3). We found that long-term treatment with 20 nM FL3 is required to increase the level of AnxA2 significantly and that the effect is transient (Figure 3A). We previously reported that Ser25 phosphorylation of AnxA2 in combination with ubiquitination and/or SUMOylation targets translationally inactive mRNP complexes to the perinuclear region, with Ser25 phosphorylation evidently triggering the other post-translational modifications (Aukrust et al., 2017). Therefore, we investigated the levels of Ser25 phosphorylated AnxA2 for up to 48 h of FL3 treatment and observed that the level of pSer25AnxA2 partially overlaps with the level of total AnxA2 with no significant differences (Figure 3).

### 3.2 FL3 increases AnxA2 protein expression and causes its re-localization to the plasma membrane

**FIGURE 4:**
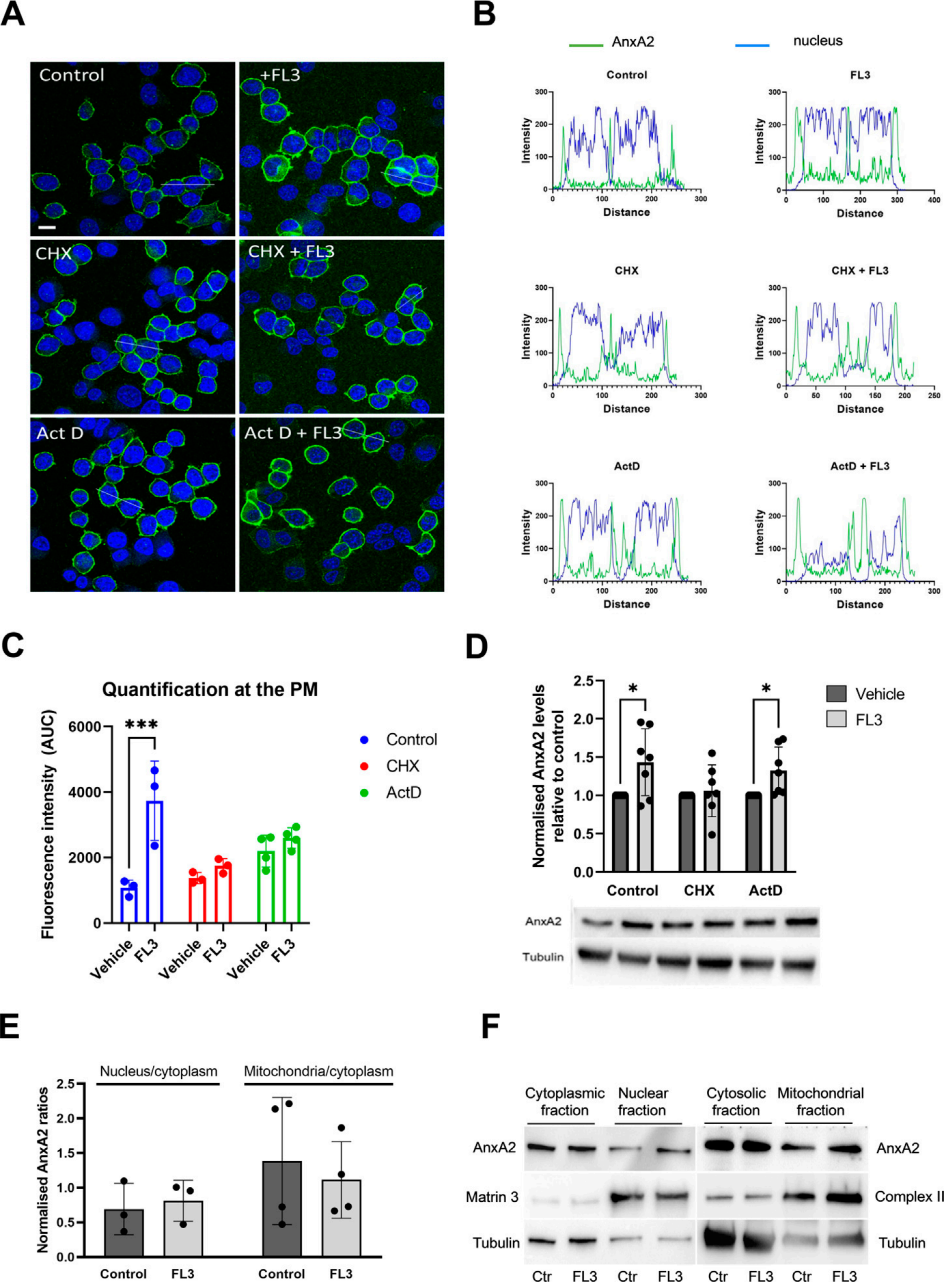
Short-term treatment with FL3 increases the expression of AnxA2 and induces its re-localization to the PM. PC12 cells were untreated (control; Ctr), treated for 2 h with 20 nM FL3 alone or in combination with cycloheximide (CHX) or actinomycin D (Act D), as indicated (Panels **A–D**). (Panel **A**) IF staining was carried out using a polyclonal antibody against AnxA2 (green). The confocal images also show DNA staining (DAPI; blue) to highlight the nuclei. Scale bar: 10 µm. (Panel **B**) shows the fluorescence intensity profiles of selected cells indicated by the white lines in (Panel **A**) with intensity of AnxA2 shown in green and nuclear staining (DAPI) in blue. Distance is measured in pixels. (Panel **C**) shows the determined areas under the peaks at the plasma membrane (PM) of the curves for AnxA2 intensity profiles shown in Panel B (*n* = 3 or 4). Panel **(D)** 15 µg of proteins derived from the corresponding lysates were separated by 10% SDS-PAGE, transferred to nitrocellulose membranes for Western blot analysis with monoclonal antibodies against AnxA2. Representative blots and the results from seven independent experiments (*n* = 7). (Panels **E** and **F**): 15 µg of proteins derived from the cytoplasmic, nuclear, cytosolic (cytoplasm without mitochondria), or the mitochondrial fractions from PC12 cells without (control; Ctr) or after treatment with 20 nM FL3 were separated by 10% SDS-PAGE and transferred to nitrocellulose membranes for Western blot analysis with monoclonal antibodies against AnxA2. Antibodies against compartment markers, i.e. the cytoplasm (tubulin; 55 kDa), nucleus (matrin 3; 125 kDa) and mitochondria (complex II; 70 kDa) were also employed as indicated. Representative blots (Panel **F**) and the results from three independent experiments (*n* = 3) (Panel **E**) are shown. (Panel **E**): The distribution of AnxA2 in the control and FL3-treated fractions as a ratio of the nucleus/cytoplasm or the mitochondria/cytoplasm AnxA2 was normalization to the loading controls (tubulin for the cytoplasmic and cytosolic fractions, matrin 3 for the nuclear fractions and complex II for the mitochondrial fractions). Statistical significance compared to control was determined by unpaired multiple *t*-test (* <0.05) (Panel **D**) and two-way ANOVA with Tukey’s multiple comparisons test (Panel **E**).

To investigate whether the initial increase in AnxA2 expression occurred at the transcriptional and/or translational levels, PC12 cells were incubated for 2 h with 20 nM FL3 in the absence or presence of actinomycin D (Act D) or cycloheximide (CHX) (Figures 4A–C). FL3 treatment of control cells for 2 h appeared to increase the expression of AnxA2 both in the cytoplasm and at the PM (Figures 4A, B, upper rows; Figure 4C). Increased levels of AnxA2 after FL3 treatment were verified by Western blot of cell lysates (Figure 4D). Treatment with CHX or Act D resulted in some increase in AnxA2 signal including stronger signal at the PM. Still, the FL3-mediated increase of AnxA2 was apparently inhibited by these treatments (Figures 4A–C). Notably, only short-term effects of these drugs were examined, since long-term treatments with the inhibitor of translation elongation (CHX), as well as the inhibitor of transcription (Act D), are likely to exert profound effects on numerous cellular processes (Rajagopalan and Gurnani, 1986). Western blot analysis of total AnxA2 in lysates derived from FL3-treated PC12 cells in the absence or presence of CHX or Act D, revealed a slight but significant increase of AnxA2 after FL3 treatment as well as FL3 combined with Act D (Figure 4D). The magnitude of the increase appears smaller in the Western blot than from the immunofluorescence (IF) staining. An explanation for this could be that the increase at the PM is more prominent in the IF images, while the overall increase as measured in the cell lysates is more modest. Overall, the data suggest a post-transcriptional effect of FL3 on AnxA2 expression.

Since the increased expression of AnxA2 after FL3 exposure led to its partial re-localization to the PM (Figure 4C), we examined whether the treatment would also lead to re-localization of the protein to nuclear and mitochondrial fractions (Figures 4E, F). AnxA2 is mainly localized to the cytoplasm, associating with endomembranes and the cytoskeleton (Hayes et al., 2004; Grieve et al., 2012). However, it also localizes to the nucleus where its different functions are regulated by phosphorylation and other post-translational modifications (Liu and Vishwanatha, 2007; Grindheim et al., 2016). Based on the presence of tubulin, the nuclear fraction also includes perinuclear membranes, as discussed previously (Aukrust et al., 2017). As shown in Figure 4E, the general increase in the expression level of AnxA2 after 2 h treatment with FL3 did not appear to lead to a significant increase in its redistribution to the nuclear fraction.

We also previously obtained evidence that some AnxA2 may be associated with mitochondria (Aukrust et al., 2017). The present experiments indicated that FL3 treatment did not alter the mitochondrial content of AnxA2 significantly (Figure 4E).

### 3.3 AnxA2 regulates the translation of its cognate mRNA

**FIGURE 5:**
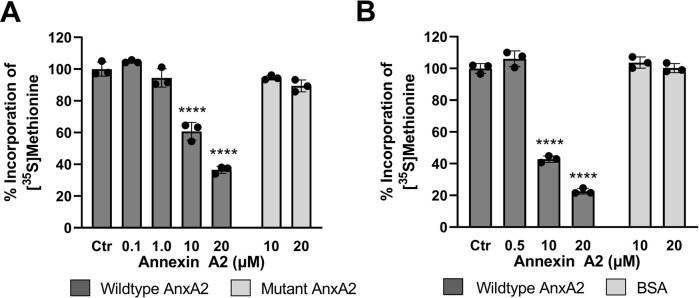
Feed-back effects of AnxA2 on the translation of its cognate mRNA in the coupled *in vitro* RRL transcription/translation system and in an *in vitro* RRL translation system. Panel **(A)** T7-driven expression of rat AnxA2 by transcription from a PCR fragment (8 ng/μL; ∼10 nM cDNA) and subsequent translation of its mRNA was performed for 60 min at 30°C in the absence (column 1) or presence of 0.1 µM (column 2), 1 µM (column 3), 10 µM (column 4) or 20 µM (column 5) of AnxA2 (Δ20AnxA2). Panel **(B)** Rat *anx*A2 mRNA was *in vitro* transcribed and 1 µg mRNA/25 µL (∼100 nM) was used in the RRL for *in vitro* translations for 60 min at 30°C in the absence (column 1) or presence of 0.5 µM (column 2), 10 µM (column 3) or 20 µM (column 4) of AnxA2 (Δ20AnxA2). Panels **(A)** and **(B)** The incorporation of [^35^S]-Met is expressed as percentage relative to the expression of AnxA2 alone (set = 100%) from its cDNA (Panel **A**) or mRNA (Panel **B**). Incorporation was measured as counts per minute (cpm) and determined using the mean value of samples withdrawn at 60 min (*n* = 3). The standard deviations are also indicated. Statistical significance compared to control was determined by one-way ANOVA with Dunnett’s multiple comparisons test (* <0.05, ** <0.01, *** <0.001, **** <0.0001).

To study the short-term effects of AnxA2 on the translation of *anx*A2 mRNA, *in vitro* coupled transcription-translations in the rabbit reticulocyte lysate (RRL) were performed. The RRL system is ideal to test the effects of exogenously added AnxA2 on translation since it lacks endogenous AnxA2 (Aareskjold et al., 2019). To define the RRL system, the monitoring of the T7-driven *in vitro* coupled transcription-translation expression of AnxA2 in the RRL system was based on PCR amplified cDNA of full-length rat *anx*A2 cDNA coding for the protein and untranslated regions (UTRs) of the mRNA with a T7 promoter (Aareskjold et al., 2019) (Figure 5A). Furthermore, the results were compared to those obtained using an *in vitro* translation system based on the RRL supplemented with an *in vitro* transcribed full-length rat *anx*A2 mRNA including the UTRs relying solely on translation (Figure 5B). In both systems, the expression of AnxA2 protein was measured as the incorporation of [^35^S]-Met into protein using end-point analysis (60 min). Previously, we have shown that AnxA2 binds to the 3′UTR and possibly also the 5′end of its cognate mRNA (Hollas et al., 2006). In the commercial RRL, endogenous mRNAs have been degraded by Ca^2+^-dependent nucleases, which have subsequently been sequestered by EGTA (Pelham and Jackson, 1976). The binding of AnxA2 to mRNA is Ca^2+^-dependent (Aukrust et al., 2006); however, adding Ca^2+^ to the RRL would be catastrophic. We have bypassed this problem by using AnxA2 lacking the first 20 amino acid residues, i.e. Δ20AnxA2, in which the RNA-binding site in the fourth domain is exposed (Strand et al., 2021), similarly as in the Ser25 phosphorylated AnxA2 in the open conformation (Ecsedi et al., 2017). The 20 most N-terminal residues (counting Met as 1) have never been observed in the obtained wild-type AnxA2 crystal structures (Rosengarth and Luecke, 2004; Raddum et al., 2015; Ecsedi et al., 2017) indicating that this N-terminus is very flexible. The most N-terminal end of the N-terminus harbors the S100A10 binding site (Réty et al., 1999) and S100A10 is not involved in the RNA-binding activities of AnxA2 (Filipenko et al., 2004; Aukrust et al., 2007). We have observed that this truncated version of AnxA2 binds RNA in the absence of Ca^2+^ in contrast to full-length AnxA2 which requires Ca^2+^ for the interaction (Vedeler and Hollas, 2000; Aukrust et al., 2007; Strand et al., 2021).

Here we show that AnxA2 regulates the translation of its cognate mRNA in a dose-response manner (Figures 5A, B). The higher levels of AnxA2 increasingly inhibit translation of its cognate mRNA. The effects were similar in the two RRL based systems tested. These results indicate that in the RRL assay, AnxA2 inversely regulates the expression of its cognate mRNA at the level of translation.

### 3.4 FL3 increases the short-term expression of AnxA2 in the *in vitro* coupled transcription-translation RRL system

**FIGURE 6:**
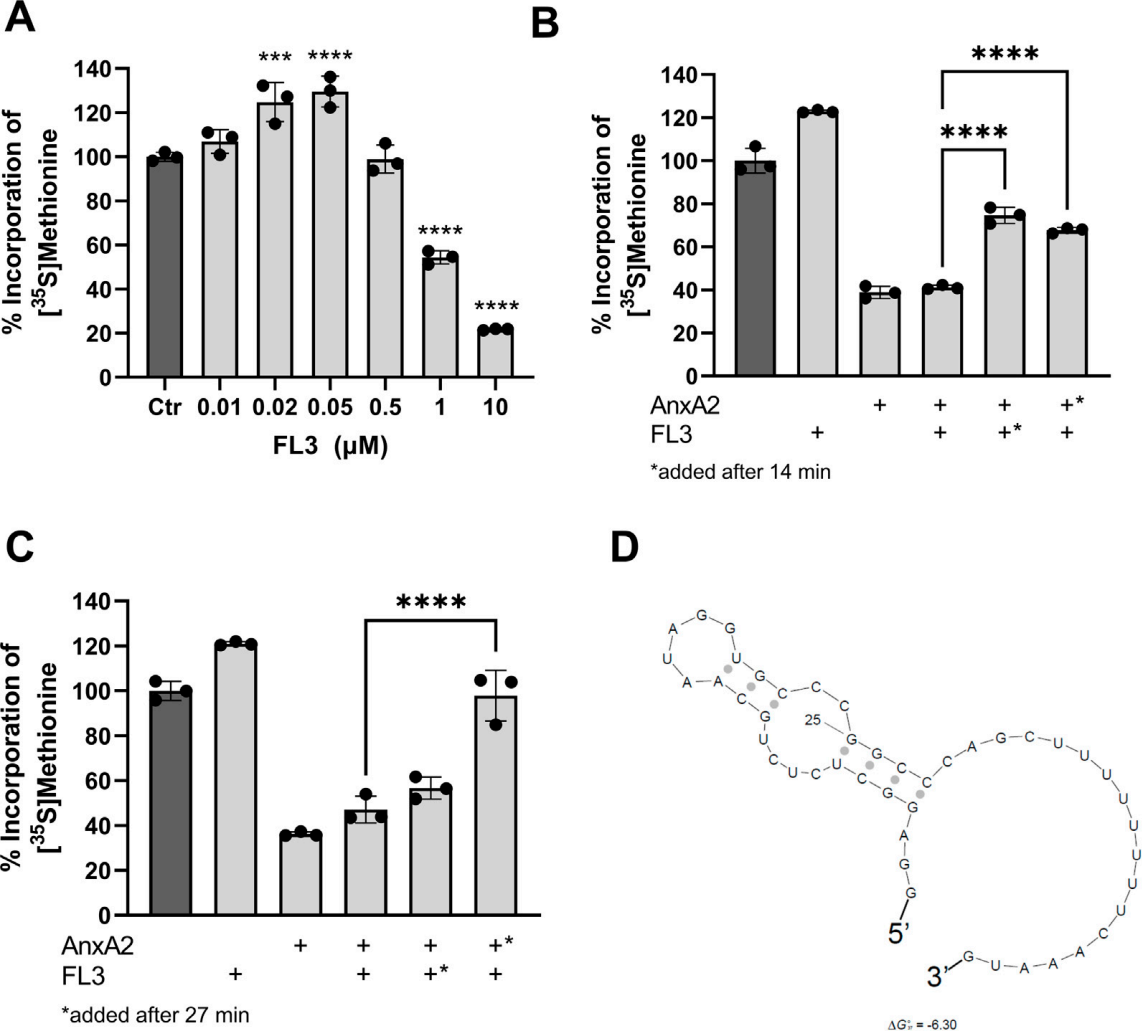
Nanomolar concentrations of FL3 increase the expression of AnxA2 and can partly relieve the inhibitory effect of 10 µM AnxA2. Panel **(A)** The T7-driven expression of rat AnxA2 from a PCR fragment (8 ng/μL) and subsequent translation of the mRNA was performed for 60 min at 30°C in the RRL system in the absence (column 1) or presence of increasing concentrations of FL3 as indicated. Significance is calculated relative to control (Ctr) (set at 100%). Panels **(B)** and **(C)** The T7-driven expression of rat AnxA2 from a PCR fragment (8 ng/μL) and subsequent translation of the mRNA was performed for 60 min at 30°C in the absence (column 1) or presence of 20 nM FL3 (column 2) or 10 µM Δ20AnxA2 (column 3) alone or added in combination (column 4) from the start. In addition, T7-driven expressions were performed in the presence of 20 µM Δ20AnxA2 before the addition of 20 nM FL3 at 14 min (Panel **B**, column 5) or 27 min (Panel **C**, column 5) or in the presence of 20 nM FL3 prior to the addition of 10 µM Δ20AnxA2 at 14 min (Panel **B**, column 6) or 27 min (Panel **C**, column 6) in the RRL system as indicated in the figure. The incorporation of [^35^S]-Met is expressed as percentage relative to the expression of AnxA2 alone from its cDNA, measured as counts per minute (cpm) and determined using the mean value of the samples withdrawn at 60 min (*n* = 3). The standard deviations are also indicated. Significance in Panels **(B)** and **(C)** of columns 5 and 6 is calculated relative to column 4 where AnxA2 and FL3 are both present from the beginning of translation. Statistical significance compared to control (Panel **A**) and to the simultaneous treatment with AnxA2 and FL3 (Panels **B** and **C**) was determined by one-way ANOVA with Dunnett’s multiple comparisons test (* <0.05, ** <0.01, *** <0.001, **** <0.0001). Panel **(D)** Prediction of the secondary structure with the lowest ΔG^o^ of the rat *anx*A2 5′UTR including the start codon using the Sfold program (ΔG^o^ = −6.3 kJ/mol) (Ding, Chan et al., 2005).

Next the effect of FL3 on the expression of AnxA2 in the *in vitro* coupled transcription-translation system was investigated. As shown in Figure 6A, short-term exposure (1 h) to FL3 increases the expression of AnxA2 in a dose-dependent manner up to a concentration of about 50 nM. Higher concentrations of FL3 inhibit the AnxA2 expression in the RRL system (Figure 6A). Thus, FL3 in nanomolar concentrations stimulates the transcription/translation of the *anx*A2 mRNA in the RRL lacking endogenous AnxA2. This concentration of FL3 has also previously been shown *in vitro* or *in vivo* as the physiologically relevant concentrations for cytotoxicity in many cancer cells (Ribeiro et al., 2012).

To address a possible interaction between AnxA2 and FL3 influencing the translation of the *anx*A2 mRNA in the RRL, 10 µM AnxA2 was allowed to inhibit the translation for 14 min or 27 min, prior to the addition of 20 nM FL3. Vice versa, 20 nM FL3 was allowed to stimulate the expression of AnxA2 for 14 min or 27 min before 10 µM AnxA2 was added to inhibit protein expression. These effects were compared with the control and the joint treatment with FL3 and AnxA2 when both were added at the start of the incubation and also related to the controls (100%) (Figures 6B, C). Evidently, when 20 nM FL3 is present together with AnxA2 from the beginning of the translation reaction, it has not the ability to counteract the inhibitory effect of 10 µM AnxA2. However, it should be noted that there is a 500-fold difference in the concentration between FL3 and AnxA2. Since both FL3 and AnxA2 inhibit translation at equimolar concentrations (10 µM) (Figure 6A), higher concentrations of FL3 were not investigated.

The addition at 27 min of 20 nM FL3 to the translation assays first inhibited by 10 µM AnxA2 had little effect on the translation of *anx*A2 mRNA, as compared to the situation when both were present from the start of translation (Figure 4C). However, the addition of FL3 after only 14 min of AnxA2-mediated inhibition relieved the inhibitory effect of the latter significantly (Figure 6B). Interestingly, 10 µM AnxA2 was not able to counteract the FL3-induced stimulation of AnxA2 expression (compare Figures 4B, C).

Flavaglines, like FL3, appear to preferentially inhibit the translation of mRNAs with highly structured and/or polypurine-rich sequences in their 5′UTRs by interacting with eIF4A (Iwasaki et al., 2016). The *anx*A2 5′UTR region is not purine-rich and does not constitute a long and very complex structure, although it appears to form a stem loop structure as determined using Sfold (Figure 6D) (Ding et al., 2005).

### 3.5 AnxA2 binds to the initiation complex eIF4F and PABP1

**FIGURE 7:**
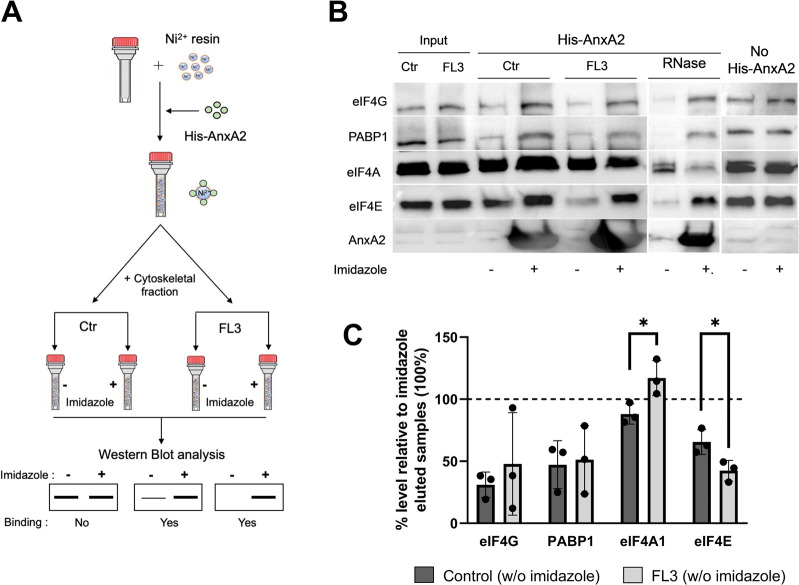
AnxA2 binds to eIF4E, eIF4G and PABP1 in the cytoskeletal fraction transiently in an RNA independent manner. Panel **(A)** A schematic representation of the pure-crude holdup method. Ni^2+^-beads were saturated with His-AnxA2 (400 µM) or left uncoated (last 2 lanes in Panel **B**). After binding and washing, the bound AnxA2 and the Ni^2+^-resin alone (last 2 lanes in Panel **B**) were incubated with the cytoskeletal fraction (Ctr or RNase treated as indicated) from untreated (control; Ctr) or FL3-treated PC12 cells (2 h) as indicated. Subsequently, each tube subjected to the different conditions was split into two tubes with equal amounts in each. The proteins with + (Panel **B**) were eluted with imidazole and the tube with—(Panel **B**) received an equal volume of 130 mM KCl buffer (which does not elute the bound AnxA2). Panel **(B)** 15 µg of proteins from the holdup assays were separated by SDS-PAGE, transferred to nitrocellulose membranes for Western blot analysis using primary antibodies against eIF4G, PABP1, eIF4A1, eIF4E and AnxA2. Representative blots are shown. Panel **(C)** Quantitation representation of the results shown in Panel **(B)** (*n* = 3). The % level of eIF4G, PABP1, eIF4A and eIF4E eluted with the KCl buffer (leaving AnxA2 on the resin) relative to imidazole eluted samples. The lower the % percentage, the more is bound to AnxA2. Statistical significance compared to control was determined by unpaired multiple *t*-test (* <0.05).

Since the employment of the monoclonal AnxA2 antibodies resulted in very modest immune precipitates, we decided to use the holdup comparative chromatographic retention assay (referred to here as the holdup assay) to detect specific proteins of the translational initiation complex possibly associating with AnxA2 (Figure 7A). Thus, this method is based on the comparative chromatographic retention of ligand-analyte pairs at equilibrium conditions (Charbonnier et al., 2006; Vincentelli et al., 2015). This infers that the (−) results on the western blots should be compared to the (+) results at the same conditions (Figure 7). In the latter case, the analyzed ligands are eluted together with His-AnxA2 by competition with high concentrations of imidazole. If a ligand binds to AnxA2 it will remain bound in buffer conditions that do not elute AnxA2 and will therefore not appear in the eluate; the (−) conditions. This method has the additional advantage of detecting transient interactions since there are no washing steps involved (Charbonnier et al., 2006; Vincentelli et al., 2015). This is an important aspect, since recent single-molecule studies have shown that the interactions between PABP, eIF4E, eIF4G could be transient (Kim et al., 2022). Furthermore, and surprisingly, these studies suggested that the spatial overlap of PABP, eIF4E and eIF4G on a single mRNA rarely occurred simultaneously challenging the present stable closed-loop model of the UTRs during translation (Kim et al., 2022). Surely, more data are needed to settle this matter.

Using the holdup method, we observed that AnxA2 binds to eIF4G, PABP1 and eIF4E from the cytoskeletal fractions derived from untreated control cells or cells treated with FL3 (Figures 7B, C). However, AnxA2 does not appear to interact with eIF4A (Figures 7B, C). Also, eIF4G appears to be sticky and also appears to bind to the resin without bound AnxA2 (Figure 7; no His-AnxA2), although it should be noted that when bound, His-AnxA2 was saturated on the resin. Note that the lower the % of a particular protein in the (−) fraction (not eluted by imidazole) is, the more is bound to AnxA2 on the Ni^2+^-resin when compared to the (+) fraction (Figure 7C). Evidently, AnxA2 binds more efficiently to eIF4E present in the cytoskeletal fraction isolated from FL3-treated cells than control cells. To investigate whether the interaction of eIF4G, PABP1 and eIF4E with AnxA2 is RNA dependent, RNase treatment was performed, and we found that all three proteins bound AnxA2 in the absence of RNA (Figures 7B, C).

### 3.6 The association of AnxA2 with eIF4F is preserved at the cap structure for a subpopulation of mRNAs translated on cytoskeletal-bound polysomes

**FIGURE 8:**
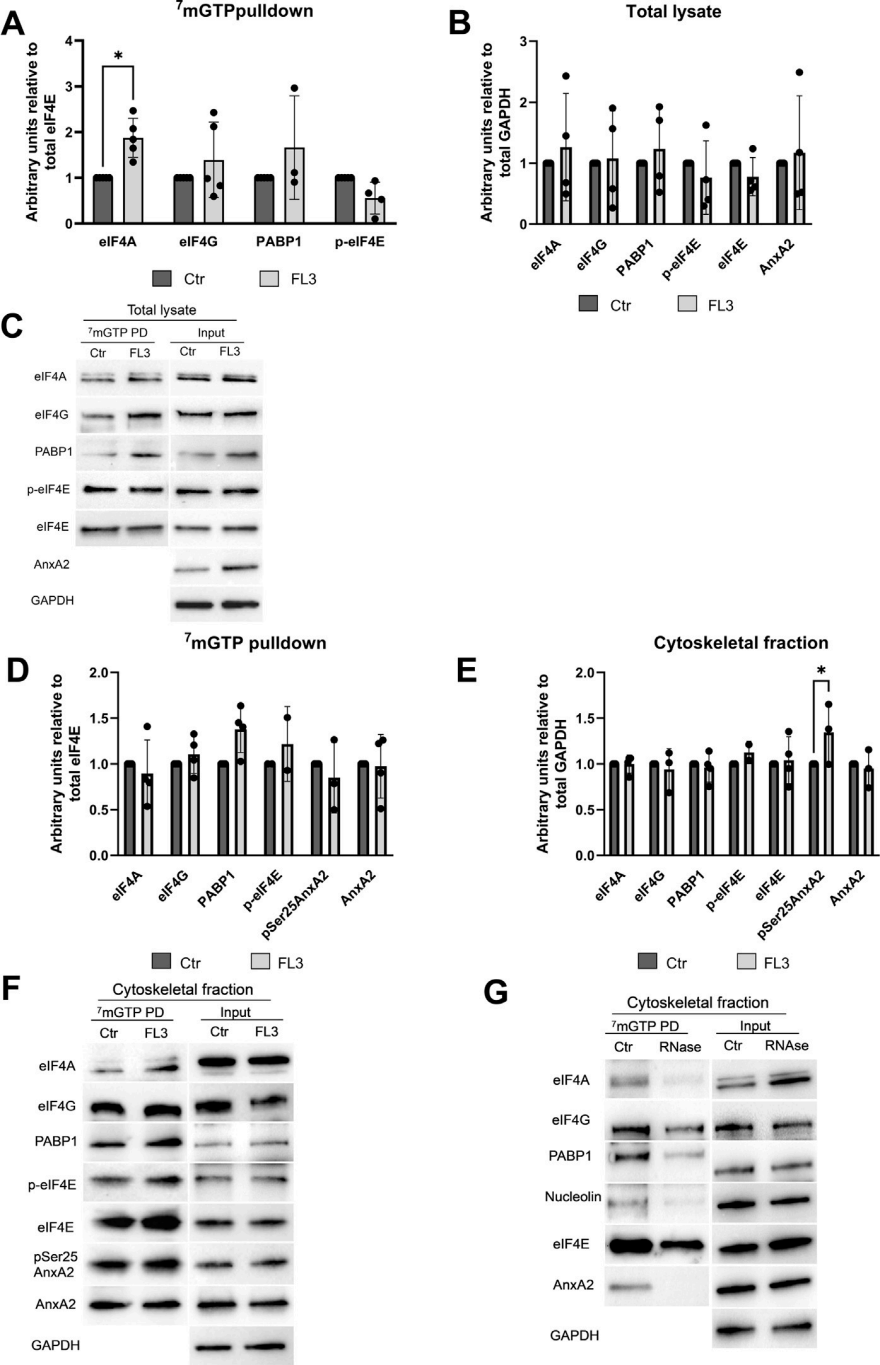
AnxA2 is associated with the cap-associated eIF4F complex from the cytoskeletal fraction but not a total lysate from PC12 cells. PC12 cells were untreated (control) or treated for 2 h with 20 nM FL3 as indicated. Total lysates (Panels **A–C**) or cytoskeletal fractions (Panels **D–G**) were obtained (1/20 of inputs) and eIF4F complexes were isolated from these fractions by m^7^GTP pulldown assays as indicated and the proteins (15 µg) were separated by 10% SDS-PAGE, transferred to nitrocellulose membranes for Western blot analysis using the indicated antibodies. GAPDH provided a loading control for lysates and cytoskeletal fractions, while eIF4E served as loading control for m^7^GTP pulldown proteins. Panel **(G)**. The cytoskeletal fraction was RNase treated before cap pulldown. Representative blots (Panels **C** and **F**) and the results from four independent experiments (*n* = 4) are shown. Proteins are expressed in arbitrary units of intensity relative to the control sample (set = 1) after normalization to the loading control GAPDH (Panels **B** and **E**) or eIF4E (Panels **A** and **D**). The standard deviations are also indicated. Statistical significance compared to control was determined by two-way ANOVA and Sidak’s multiple comparisons test (* <0.05).

Subsequently, to investigate in greater detail the effects of AnxA2 and FL3 on the regulation of translation initiation, cap pulldown experiments were employed. Eukaryotic mRNAs contain a cap structure, m^7^GpppN, at the 5′end where N is any nucleotide and the initiation factor eIF4F binds to the cap structure via the eIF4E subunit (Pelletier and Sonenberg, 2019). As described above, AnxA2 and FL3 apparently counteracted in the RRL to some degree each other’s influence on the translation of the *anx*A2 mRNA (Figure 6). Furthermore, since FL3 binds directly to eIF4A and enhances its RNA binding (Chu et al., 2019), we performed m^7^GTP affinity purification (cap pulldown assays) for the detection of the three protein components of the eIF4F complex - eIF4A, eIF4G and eIF4E (including its phosphorylated form)—in the total lysate of PC12 cells. The goal was to investigate the possible presence of AnxA2 in the initiation complex and the effect of FL3 on complex formation of these proteins and also PABP1 in PC12 cells (Figure 8). AnxA2 was previously found to associate with its cognate mRNA when using *in vitro* transcribed full-length *anx*A2 mRNA as a bait to capture AnxA2 in the cytoskeletal fraction of PC12 cells (Aukrust et al., 2017).

Treatment with FL3 for 2 h significantly increases the proportion of eIF4A found in the cap pulldown complexes from total lysates relative to eIF4E (Figures 8A, C). Only negligible amounts of AnxA2 were detected in the cap pulldown complexes from total lysates. AnxA2 was only faintly detected in a few cap pulldown complexes from lysates from control cells, suggesting that in lysates derived from FL3-treated cells this multifunctional protein interacts with other proteins, since the 2 h FL3 treatment resulted in increased expression of AnxA2 (Figure 3).

Indeed, affinity-purification of cap-associated complexes from the cytoskeletal fraction resulted in the detection of AnxA2 and its Ser25 phosphorylated form, as well as PABP1 in these complexes (Figures 8D–F), supporting previous results on the association of AnxA2 with a subpopulation of specific mRNAs translated on cytoskeletal polysomes (Vedeler and Hollas, 2000; Hollas et al., 2006; Vedeler et al., 2012). The Ser25 phosphorylation of AnxA2 in combination with other PTMs has previously been implicated in sequestering translationally inactive mRNAs (Aukrust et al., 2017). We next tested whether AnxA2 is found in the cap pulldown complexes due to its binding to specific mRNAs as previously reported (Mickleburgh et al., 2005; Hollas et al., 2006; Vedeler et al., 2012). After the pretreatment of the cytoskeletal fraction with RNase, there was clearly no binding of AnxA2 to the cap complex (Figure 8G) and the results indicate that the interactions of AnxA2, nucleolin, eIF4A and partly PABP1 with eIF4E is RNA dependent (Figure 8G).

## 4 Discussion

### 4.1 FL3 transiently increases the expression of AnxA2 and Ser25 phosphorylated AnxA2

AnxA2 is a multifunctional protein, which is frequently deregulated–in most cases undergoing upregulation–in many types of cancers including those of the nervous system (Maule et al., 2016; Christensen et al., 2018). The overexpression of AnxA2 in cancers often correlates with resistance to treatment, metastasis, and thus poor prognosis (Christensen et al., 2018). On the other hand, AnxA2 peptides presented by MHC class II-positive cancer cells activate antigen-specific T cells and consequently produce an immune response that can be exploited in immunotherapy (Heinzel et al., 2001; Zheng and Jaffee, 2012; Weyd, 2016). FL3 is a synthetic flavagline with potent anticancer effects (Thuaud et al., 2009) having eIF4A as one of its primary targets but also targets other helicases such as DDX3 (Chen et al., 2021), while AnxA2 is involved in regulation of mRNA transport and translation of specific mRNAs including its cognate mRNA (Vedeler and Hollas, 2000; Mickleburgh et al., 2005; Vedeler et al., 2012; Strand et al., 2021). We found that FL3 at 20 nM increases the expression of AnxA2 transiently (Figure 3A), and that the level of Ser25 phosphorylated AnxA2 follows the expression of the protein (Figure 3B). Others have shown that treatment with another flavagline, Silvestrol, increases the amount of *anx*A2 mRNA in polysomes by about 10% after 24 h (Ho et al., 2021) (Supplementary Material). AnxA2 is a long-lived protein with a half-life of about 20–45 h depending on cellular conditions (Cuervo et al., 2000), indicating a slow turnover. A relatively small induction by FL3 on AnxA2 expression over time together with slow AnxA2 turnover may explain our results.

It is possible that the reduced expression of AnxA2 upon long-term FL3 exposure results from the sequestration of transitionally inactive mRNP complexes/granules by pSer25 phosphorylated AnxA2 and/or their silencing during transport to the site of translation (Aukrust et al., 2017). Flavaglines, such as FL3 preferentially inhibits the translation of mRNAs with highly purine-rich structured 5′UTRs that require eIF4A activity. It has also been reported that some mRNAs are more actively translated upon flavagline treatment (Ho et al., 2021). Thus, it was suggested that flavaglines may both inhibit and activate different translation factors and remodel the translation machinery (Ho et al., 2021). It is possible that long term (≥72 h) treatment of PC12 cells with FL3 would lead to inhibition of translation of the *anx*A2 mRNA. However, under these conditions the cells changed morphologically and rounded up. This effect has also been observed previously as an effect of other flavaglines. As a simultaneous RhoA activation was seen, the effect could be related to changes in cytoskeletal dynamics (Ho et al., 2021).

### 4.2 FL3 and AnxA2 modulate the translation of *anx*A2 mRNA in the RRL system

FL3 treatment led to an increase in AnxA2 expression in PC12 cells, and also involves a partial re-localization of the protein to the PM (Figure 4). Using the RRL assay we also found that AnxA2 inhibits the expression of its cognate mRNA at the level of translation (Figure 5). To evaluate whether the concentrations of AnxA2 used to analyze its effect on the regulation of translation of its cognate mRNA demonstrated in the RRL have any *in vivo* relevance, we previously carried out calculations showing that the approximate concentration of AnxA2 in PC12 cells is about 6-7 μM) (Strand et al., 2021). Moreover, stimulation of the cells with nerve growth factor (NGF) results in a 3- to 14- fold increase in the concentration of AnxA2 (Fox et al., 1991; Jacovina et al., 2001). The *anx*A2 mRNA is translated on cytoskeleton-bound polysomes in the perinuclear area (Veyrune et al., 1996; Vedeler and Hollas, 2000; Hollas et al., 2006) where the concentration of AnxA2 is believed to be lower due to the typical enrichment of the protein in the cortical region underneath the PM (Gerke and Moss, 2002). Thus, the local subcellular concentrations of AnxA2 may differ substantially and only a distinct pool of the protein is involved in transport/translation of specific mRNAs. However, the feed-back mechanism of AnxA2 in the regulation of the translation of its cognate mRNA appears to occur at physiologically relevant concentrations of the protein (0.1–20 µM) as investigated in the RRL system (Figure 5). AnxA2 was also found to reduce the expression of proprotein convertase subtilisin kexin type 9 (PCSK9) at the level of translation (Ly, Luna Saavedra et al., 2014), indicating its ability to inhibit the translation of certain mRNAs. In addition, there are several other examples of regulatory feed-back mechanisms during translation exerted by proteins binding to their cognate mRNAs (Pullmann et al., 2007). Since the binding of AnxA2 to mRNA is Ca^2+^-dependent (Aukrust et al., 2007), the concentration of Ca^2+^ is important for the interaction. Our previous SPR data suggest that there is an initial fast Ca^2+^-dependent electrostatic interaction between AnxA2 and RNA, possibly followed by conformational changes in both interacting components (Aukrust et al., 2007). This could result in the interaction of AnxA2 with other ligands than those associating with its non-RNA bound form.

The RRL system was chosen primarily since it lacks endogenous AnxA2 (Strand et al., 2021) although this system is not ideal due to the low level of cap-dependent translation (Svitkin et al., 1996). However, it was used as an initial assay to investigate in particular the mutual effects of AnxA2 and FL3 on the translation of the *anx*A2 mRNA (Figures 5, 6). The apparent short-term FL3-mediated increase in the expression of AnxA2 in PC12 cells at the level of translation at 20 nM (Figures 3, 4) was corroborated by the results obtained using the RRL system (Figure 6A). The addition of FL3 after only 14 min (but not after 27 min) of AnxA2-mediated inhibition, relieved the inhibitory effect of the latter significantly (Figure 6B), while 10 µM AnxA2 was not able to inhibit the FL3-induced stimulation of AnxA2 expression (compare Figures 6B, C). This suggests that FL3 may mask a binding site in a translation initiation factor or another ligand that interacts directly or indirectly with AnxA2. Since there is a 500-fold difference in concentration, a direct interaction between AnxA2 and FL3 is unlikely.

The apparent effect of FL3 on AnxA2-mediated regulation of translation could occur via the translation initiation complex eIF4F, since FL3 has been reported to affect its formation (Boussemart et al., 2014). eIF4F is a trimeric complex that includes eIF4A (an RNA helicase), eIF4E (a cap-structure-binding protein) and eIF4G, a scaffolding protein required for the recruitment of other translation factors and the 40S ribosome (Pelletier and Sonenberg, 2019). AnxA2, on the other hand, has been reported to associate with PABP (Filipenko et al., 2004), another protein that is important for initiation of translation by mediating crosstalk between the 5′- and 3′-UTRs via eIF4G (Derry et al., 2006). Both eIF4A and PABP1 bind to eIF4G; however, by interacting with different sites (Pelletier and Sonenberg, 2019).

### 4.3 AnxA2 derived from the cytoskeletal fraction is associated with initiation factors

AnxA2 binds to the 3′UTR of its cognate mRNA and the c-*myc* mRNA in a Ca^2+^-dependent manner (Mickleburgh et al., 2005; Hollas et al., 2006). Furthermore, it has previously been shown that AnxA2 interacts with PABP1 (Filipenko et al., 2004), which in turn interacts with the eIF4G subunit of the initiation complex eIF4F (Pelletier and Sonenberg, 2019). The NS5A nonstructural protein of hepatitis C virus (HCV) was shown to be involved in mRNA translation (He et al., 2001) by binding to the eIF4F initiation complex (George et al., 2012). Interestingly, it has also been shown that AnxA2 binds to HCV 5S5B with high affinity and that both proteins associate with different specific RNAs (Solbak et al., 2017), indicating yet another link between AnxA2 and initiation of translation.

By using the holdup method which can identify transient interactions, we found that AnxA2 apparently binds to eIF4G, PABP1 and eIF4E and that FL3 possibly influences the interaction of eIF4E with AnxA2 (Figures 7B, C). RNase treatment of the cytoskeletal fraction before binding to immobilized AnxA2 did not affect the interaction of eIF4G, PABP1 and eIF4E with AnxA2 indicating that they bound in an RNA-independent manner, at least transiently. eIF4G appears to also interact with the nickel resin (Figures 7B, C). The significance of the interaction between AnxA2 and PABP1 as well as eIF4E and possibly eIF4G subunits of eIF4F as detected by the holdup method (Figure 7) is unknown but appears to be related to initiation of translation.

However, both eIF4E and eIF4A (as well as PABP1) bind to eIF4G (Marcotrigiano et al., 2001). Possibly the binding of FL3 to eIF4A (Boussemart et al., 2014) induces a conformational change in eIF4A, which in turn affects the interaction between eIF4A and eIF4G to modulate the interaction between eIF4G and eIF4E, rendering the eIF4E more accessible to AnxA2. This is in line with the holdup data suggesting that AnxA2 binds more efficiently to eIF4E present in the cytoskeletal fraction isolated from FL3-treated cells than in control cells. Alternatively, the binding of the small ribosomal subunit to the initiation complex could be hampered.

It should be noted that the apparent binding of AnxA2 to several of the subunits of eIF4F may involve the whole eIF4F complex but not to the individual subunits of eIF4F as such since they interact with each other with high affinity (Hilbert et al., 2010; Gu et al., 2021). The lack of apparent binding of AnxA2 to eIF4A and the effect of FL3 on the interaction between eIF4E and AnxA2 would argue against this possibility.

Enhanced cap pulldown of eIF4A from a total lysate from PC12 cells by the m^7^GTP cap analogue after 2 h treatment with FL3 may indicate an increased eIF4F complex formation for active translation of specific mRNAs or could also indicate that eIF4A is trapped in the initiation complexes. The effect of FL3 on the association of p-eIF4E with the cap pulldown proteins was also analyzed since phosphorylation of the Ser209 site of eIF4E has been correlated with the initiation of translation of certain mRNAs (Uttam et al., 2018). Apparently, FL3 has no or little impact on the phosphorylation of Ser209 of eIF4E (Figure 8). AnxA2 did not appear to be an integral part of cap pulldown complexes from total lysates (Figures 8A–C) while it is a component of cap pulldown complexes from the cytoskeletal fraction (Figures 8D–F), indicating that AnxA2 associates with a subpopulation of mRNAs (Vedeler et al., 2012). Furthermore, Ser25 phosphorylated AnxA2 is part of the cytoskeletal cap pulldown complexes and FL3 increases the fraction of AnxA2 in the cytoskeletal fraction which is Ser25 phosphorylated.

The holdup experiments indicated that there is at least a transient interaction of AnxA2 with subunits of the eIF4F and/or PABP1 which is not mediated by the binding to mRNA (Figure 7). Cap pulldown of proteins is dependent on a more stable interaction. We again performed RNase treatment of the cytoskeletal fraction before performing cap pulldown experiments and included detection of nucleolin which binds both directly and indirectly to mRNA (Fähling et al., 2006) (F). From these experiments it is clear that a more stable interaction of AnxA2 with eIF4F and/or PABP1 is RNA-mediated (Figure 8G). PABP1 binds to the poly(A) tail; it is involved in the circularization of mRNA by binding to eIF4G for active translation (Sonenberg and Hinnebusch, 2009) and has previously been found in cap pulldown complexes (Buxadé et al., 2008).

The mechanistic details of these novel molecular interactions are still not completely evident. However, it is tempting to speculate that AnxA2 bound to the 3′UTR (Mickleburgh et al., 2005; Fähling et al., 2006; Hollas et al., 2006) inhibits translation by binding to PABP1, thus preventing the crosstalk between the 5′- and the 3′-UTRs. Another possibility is related to the transient association of AnxA2 with eIF4F, in particular eIF4E. AnxA2 harbors two putative eIF4E-binding sites in domain IV of its core structure (Mader et al., 1995), namely Y (270)FADRLY (in helix A) and Y (329)QKAlLY (in helix D), and appears to bind to the eIF4E subunit of eIF4F (Figure 7). Furthermore, the mRNA-binding site of AnxA2 is also present in domain IV of AnxA2, encompassing helices C-D (Aukrust et al., 2007). Thus, AnxA2 would not be expected to bind RNA and eIF4E simultaneously since mRNAs are large. A possible scenario could be that AnxA2 binds transiently to eIF4E and PABP1 simultaneously and that this interaction is strengthened by RNA binding, resulting in inhibition of translation initiation. To ensure that only specific mRNAs are affected, AnxA2 may inhibit translation as a dimer. Accordingly, one of the AnxA2 subunits could bind in a Ca^2+^-dependent manner to the 3′UTR of specific mRNAs, such as the *anx*A2 mRNA, and to PABP1, while the other subunit could bind eIF4E. The resolution of the structure of Ca^2+^-bound AnxA2 as a dimer (pdb: 1XJL) (Rosengarth and Luecke, 2004) indicates that the domain III of each AnxA2 monomer is involved in the dimerization, leading to the exposure of the RNA-binding sites in helices C-D of domain IV at the opposite side of the dimerization site. Thus, AnxA2 could be envisioned to play a role in Ca^2+^-regulated synaptic translation.

It is also possible that AnxA2 binds as a monomer to the 3′UTR and/or possibly also to eIF4E to tether RNA to vesicles similarly to AnxA11 (Liao et al., 2019). Using the crystal structure of the AnxA2 dimer (pdb: 1XJL; (Rosengarth and Luecke, 2004)), its width was measured as ∼7 nm. Furthermore, using AlphaFold, the length and width of eIF4G were measured as ∼18 nm and ∼12 nm, respectively (https://alphafold.ebi.ac.uk/entry/Q04637) (Jumper et al., 2021). The molecular weights of AnxA2 and PABP1 are 39 kDa and 72 kDa, respectively. Taking these sizes into consideration, the formation of an AnxA2 dimer may be favored. These are issues to settle in future experiments.

Nonetheless, in conclusion, the key findings of these studies demonstrate that a pool of AnxA2 is involved in the translation of specific mRNAs. Accordingly, we show that AnxA2 regulates the translation of its cognate mRNA. Thus, AnxA2 evidently associates with the initiation complex eIF4F and may do so by interacting with PABP1 and the eIF4E subunit of the eIF4F complex to inhibit translation by preventing the formation of the full eIF4F complex. We also provide evidence that short-term treatment with FL3 stimulates the translation of *anx*A2 mRNA and modulates the interaction of AnxA2 with the initiation complex.

## Data Availability

The original contributions presented in the study are included in the article/Supplementary Material, further inquiries can be directed to the corresponding author.
