# Supplementary figures and images for: The flavagline FL3 interferes with the association of Annexin A2 with the eIF4F initiation complex and transiently stimulates the translation of annexin A2 mRNA
Source: Front Cell Dev Biol. 2023 May 12;11:1094941. doi: 10.3389/fcell.2023.1094941 (PMC10214161; doi:10.3389/fcell.2023.1094941)

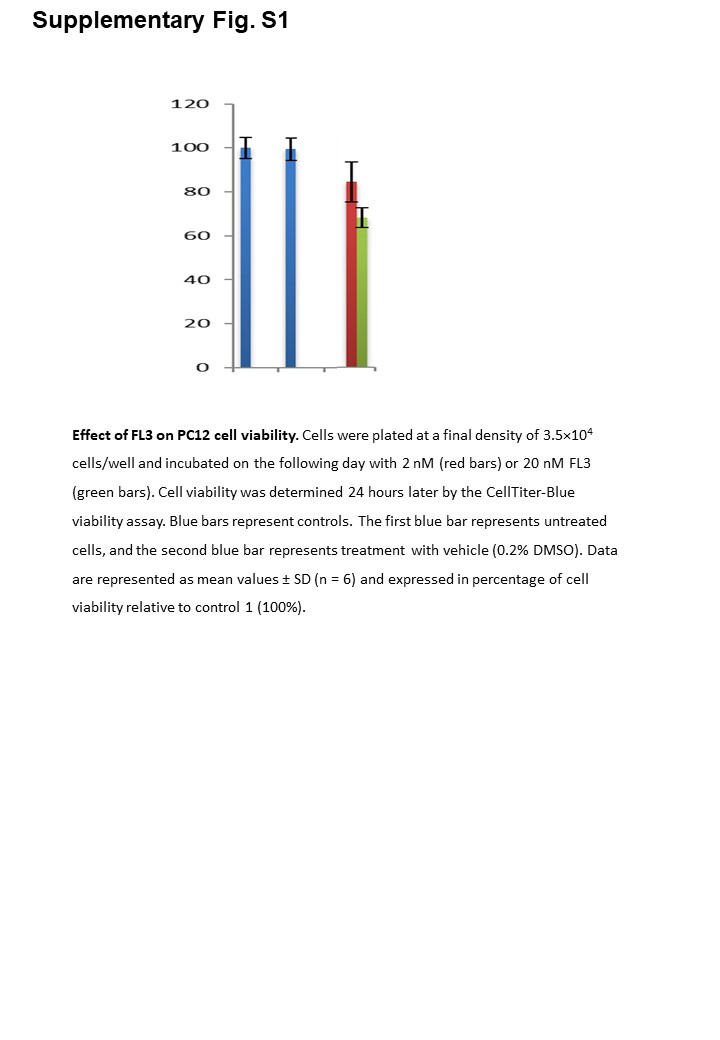

Supplement: Supplementary file 1 [file Image2.JPEG]
